# Risk factors for postoperative renal dysfunction following open surgical repair of abdominal aortic aneurysms retrospective analysis

**DOI:** 10.18632/oncotarget.22049

**Published:** 2017-10-25

**Authors:** Zuowei Wu, Ding Yuan, Jichun Zhao, Bin Huang

**Affiliations:** ^1^ West China Medical School of Sichuan University, Chengdu 610041, Sichuan Province, China; ^2^ Department of Vascular Surgery, West China Hospital, Chengdu 610041, Sichuan Province, China

**Keywords:** abdominal aortic aneurysm, renal failure, risk factor, laparotomy, scoring system

## Abstract

**Objectives:**

To identify the risk factors for postoperative renal dysfunction after open surgical repair (OSR) of abdominal aortic aneurysms (AAAs) and to establish a scoring system.

**Results:**

The overall 30-day mortality rates were 22.0%(ruptured) vs 2.6% (unruptured), respectively. For the ruptured group, the independent risk factors were hemodynamic instability (*P* = 0.002) blood loss >1 L (*P* = 0.041) and preoperative creatinine >150 μmol/L (*P* < 0.001). By contrast, for the unruptured group, factors were smoking (*P* = 0.028), blood loss >1 L (*P* = 0.018), and antihypertensive drugs (*P* < 0.001). The areas under the curve of the WCRDA scoring system are 0.794 (95% confidence interval (CI) 0.686–0.902, *P* < 0.001) and 0.811 (95% CI 0.691–0.932, *P* < 0.001) for the ruptured and unruptured groups, respectively.

**Conclusions:**

Hemodynamic instability, blood loss >1 L and Hb <90 g/L are independent risk factors for postoperative renal dysfunction following rAAA OSR, whereas smoking, blood loss >1 L, and antihypertensive drugs are independent risk factors. WCRDA performs well in predicting postoperative renal dysfunction.

**Materials and Methods:**

287 patients from the Vascular Department of West China Hospital, Sichuan University, who were planned to perform OSR for AAA from November 2003 to January 2017. 274 patients underwent OSR for AAA were finally included in the study. A total of 118 patients had ruptured AAA and 156 unruptured AAA.

The patients were divided into the ruptured and unruptured groups. Logistic regression was used to identify the independent risk factors for postoperative renal dysfunction. The receiver operating characteristic curve was used to evaluate the scoring system.

## INTRODUCTION

Despite authors [1] having reported positive results with endovascular aneurysm repair (EVAR) for abdominal aortic aneurysm (AAA), laparotomy or open surgical repair (OSR) is still performed in many situations because not all cases of AAA allow for a standard EVAR. However, elective OSR has been associated with a 30-day mortality rate of 3.0%–8.2% [2, 3]. Most specific tools [4] for predicting preoperative risk consider preoperative renal function. In addition, postoperative renal dysfunction is a significant complication leading to a high mortality rate [5]. These models only refer to the relationship between preoperative renal dysfunction and the prognosis, or postoperative renal dysfunction and the mortality. However, we do not know which factors are the risk factors for renal dysfunction, which patients need a kidney protection and whether kidney protection would be helpful to improve the prognosis. A study [6] suggest that the prevalence rates and mortality of AAA are not same in different countries or races. Another prospective multicenter study [7] has identified some risk factors for postoperative renal dysfunction. However, only elective operations were included, Chinese or Asian populations were not involved, and intra- and postoperative variables such as postoperative creatinine, urine volume, intraoperative blood loss, plaque and thrombosis were all absent. In addition, the study did not mention whether the risk factors for renal dysfunction in patients with ruptured AAA (rAAA) are the same.

The objectives of the current study were as follows: to identify the risk factors for renal dysfunction in patients who underwent OSR for ruptured and unruptured AAAs and to establish a scoring system for West China renal dysfunction after AAA or rAAA OSR (WCRDA) for further analysis of postoperative renal dysfunction prevention.

## RESULTS

### Patient characteristics, outcomes, and univariate analysis of risk factors for postoperative renal injury

Patient characteristics and postoperative renal outcomes are shown in Table 1. During the study, 274 patients successfully underwent OSR. A total of 118 were proven to have rAAAs, whereas 156 did not. The median age of the ruptured group was 58.2 ± 12.1 years, with the majority being female (*n* = 68; 57.6%), whereas that of the unruptured group was 57.8 ± 13.7 years, with the majority being male (*n* = 93; 59.6%). Among patients with juxta-/supra renal AAA, 4 in ruptured group and 6 in unruputured group were found to have had supra renal clamping. In the ruptured group, candidate univariate (*P* < 0.100) analysis of risk factors included age >65 (*P* = 0.021), preoperative creatinine >150 μmol/L (*P* < 0.000), hemodynamic instability (*P* < 0.001), smoking (*P* = 0.075), Hb <90 g/L (*P* = 0.002), and blood loss >1 L (*P* = 0.041). However, for the unruptured group, it included age >65 (*P* = 0.063), preoperative creatinine >150 μmol/L (*P* = 0.051), smoking (*P* = 0.029), antihypertensive drugs (*P* < 0.001), and blood loss >1 L (*P* = 0.049).

**Table 1:** Patients’characteristics

Characteristics	Ruptured *n* = 118	Post-operative renal dysfunction %		Odds ratio (95%CI)	P (X2test)	Unruptured *n* = 156	Post-operative renal dysfunction %		Odds ratio (95%CI)	P (X2test)
Gender Male Female	50 68	15 12	30.0% 17.6%	2.000 (0.839–4.767)	0.114	93 63	10 5	10.8% 7.9%	1.398 (0.454–4.304)	0.558
Age >65 ≤65	32 86	12 15	37.5% 17.4%	2.840 (1.147–7.033)	0.021	50 106	8 7	16.0% 6.6%	2.694 (0.918–7.906)	0.063
Symptomatic AAA Yes No	118 0	27 0	22.9% NA	NA	NA	32 124	2 13	6.3% 10.5%	0.569 (0.122–2.662)	0.469
Hemodynamic instability Yes No	31 87	15 12	48.4% 13.8%	5.859 (2.309–14.872)	0.000	0 156	0 15	NA 9.6%	NA	NA
Preoperative creatinine >150 umol/L ≤150 umol/L	12 106	9 18	75.0% 17.0%	14.667 (3.611–59.569)	0.000	2 154	1 1	50.0% 0.6%	10.000 (0.593–168.732)	0.051
Unconsciousness Yes No	10 108	2 25	20.0% 23.1%	0.830 (0.165–4.164)	0.821	2 154	0 15	0.0% 9.7%	NA	0.642
Smoking Yes No	40 78	13 14	32.5% 17.9%	2.201 (0.914–5.300)	0.075	63 93	10 5	15.9% 5.4%	3.321 (1.077–10.242)	0.029
History of respiratory diseases Yes No	14 104	2 25	14.3% 24.0%	0.527 (0.110–2.514)	0.415	19 137	3 12	15.8% 8.8%	1.953 (0.497–7.671)	0.330
Antihypertensive drugs Yes No	15 103	5 22	33.3% 21.4%	1.841 (0.570–5.946)	0.302	25 131	8 7	32.0% 5.3%	8.336 (2.682–25.909)	0.000
Juxta-/supra renal AAA Yes No	7 111	2 25	28.6% 22.5%	1.376 (0.252–7.526)	0.712	10 146	1 14	10.0% 9.6%	1.048 (0.123–8.888)	0.966
AAA diameter >6cm ≤6cm	62 11	16 4	25.8% 36.4%	0.754 (0.278–2.046)	0.578	104 52	8 7	7.7% 13.5%	0.536 (0.183–1.569)	0.249
Mural thrombus Yes No	38 80	8 19	21.1% 23.8%	0.856 (0.336–2.180)	0.744	69 87	7 8	10.1% 9.2%	1.115 (0.383–3.242)	0.842
Atherosclerotic plaque Yes No	41 77	12 15	29.3% 19.5%	1.710 (0.711–4.115)	0.228	71 85	8 7	11.3% 8.2%	1.415 (0.487–4.114)	0.522
Hb <90 g/L ≥90 g/L	30 88	13 14	43.3% 15.9%	4.042 (1.610–10.150)	0.002	18 138	3 12	16.7% 8.7%	2.100 (0.0.532–8.295)	0.281
Blood loss >1 L ≤1 L	54 64	17 10	31.5% 15.6%	2.481 (1.023–6.018)	0.041	32 124	6 9	18.8% 7.3%	2.949 (0.965–9.012)	0.049
Study period First half Second half	59 59	13 14	22.0% 23.7%	1.101 (0.466–2.600)	0.827	78 78	5 10	6.4% 12.8%	2.147 (0.698–6.601)	0.174
Total	118	27	22.9%			156	15	9.6%		
Postoperative death ratio	22.0%					2.6%				

a: CI Confidence interval; b: AAA Abdominal aortic aneurysm; c: Hb Hemoglobin.

### Multivariate analysis of risk factors for postoperative renal injury

Independent risk factors for postoperative renal dysfunction of the two groups were as follows: For the ruptured group, they were hemodynamic instability (odds ratio (OR) = 4.371, *P* = 0.005), preoperative creatinine >150 μmol/L (OR = 13.508, *P* = 0.001) and blood loss >1 L (OR = 3.081, P = 0.039). For the unruptured group, they were smoking (OR = 4.055, *P* = 0.028), antihypertensive drugs (OR = 11.180, *P* < 0.001), and blood loss >1 L (OR = 4.956, *P* = 0.018).

### Patient's postoperative complications, prognosis, and hospital information

The overall 30-day mortality rates were 22.0% and 2.6% for the ruptured and unruptured groups, respectively. Average length of postoperative stay, complications of Clavien–Dindo classification [8], intensive care unit (ICU) time, and information of each group are shown in Table 2. Postoperative renal dysfunction was found in 27 (22.9%) and 15 (9.6%) patients for the ruptured and unruptured groups, respectively (OR = 2.789, 95% confidence interval (CI) = 1.407–5.527). In total, seven patients needed renal replacement therapy, five (18.5% of renal dysfunction) in the ruptured group and two (13.3% of renal dysfunction) in the unruptured group. One patient in the ruptured group who received dialysis died 3 days after the operation.

**Table 2:** Patient's outcome

Characteristics		Ruptured (*n* = 118)	Unruptured (*n* = 156)	Total (*n* = 274)
		patients (% that ruptured)	patients (% that unruptured)	patients (% that all patients)
Death ratio		26 (22.0%)	4 (2.6%)	30 (10.9%)
Post-operative renal dysfunction		27 (22.9%)	15 (9.6%)	42 (15.3%)
Complications (Clavien-Dindo classification)	1	35 (29.7%)	31 (19.9%)	66 (24.1%)
	2	21 (17.8%)	30 (19.2%)	51 (18.6%)
	3	9 (10.2%)	7 (4.5%)	16 (5.8%)
	4	8 (6.8%)	12 (7.7%)	20 (7.3%)
Renal replacement therapy needed		5 (4.2%)	2 (1.3%)	7 (2.6%)
ICU time (hours)		110.6 ± 128.4	51.8 ± 50.1	80.2 ± 100.4

a: ICU intensive care unit

### Establish and performance of WCRDA risk estimation models

The postoperative renal dysfunction prediction model is shown in Table 3. The scoring system was derived from the logistics regression equation as follows: for rAAA patients, X = (1.475 if hemodynamic instability) + (2.603 if preoperative creatinine >150 μmol/L) + (1.125 if blood loss >1 L) – 2.702; as for AAA patients, X = (1.400 if smoking) + (2.414 if antihypertensive drugs) + (1.601 if blood loss >1 L) – 4.159. The probability of kidney dysfunction = exp (X)/(1 + exp (X)). The parameters in the equation stem from the logistic regression coefficients value. The contents of the two scoring systems are different. The formula is calculated by logistic regression analysis and transformed into the scoring system approximately. In the ruptured group, 1 score is given for hemodynamic instability or blood loss >1 L, and 2 for preoperative creatinine >150 μmol/L. The postoperative renal dysfunction probabilities were as follows: 0 = 6%, 1 = 20%, 2 = 48%, 3 = 77%, and 4 = 92%. Similarly, 1 score is given for smoking or blood loss >1 L, and 2 for antihypertensive drugs. For the unruptured group, the probabilities were as follows: 0 = 2%, 1 = 6%, 2 = 24%, 3 = 41%, and 4 = 78%.

**Table 3:** Post-operative renal dysfunction prediction model (WCRDA score)

for ruptured	score	for unruptured	score
Hemodynamic instability	+1	Smoking	+1
Blood losss >1 L	+1	Blood losss >1 L	+1
Preoperative creatinine >150 umol/L	+2	Antihypertensive drugs	+2
WCRDA score for ruptured	Predicted risk	for unruptured	Predicted risk
0	6%	0	2%
1	20%	1	6%
2	48%	2	24%
3	77%	3	41%
4	92%	4	78%

a: WCRDA: West China renal dysfunction after AAA or rAAA OSR

**Figure 1:**
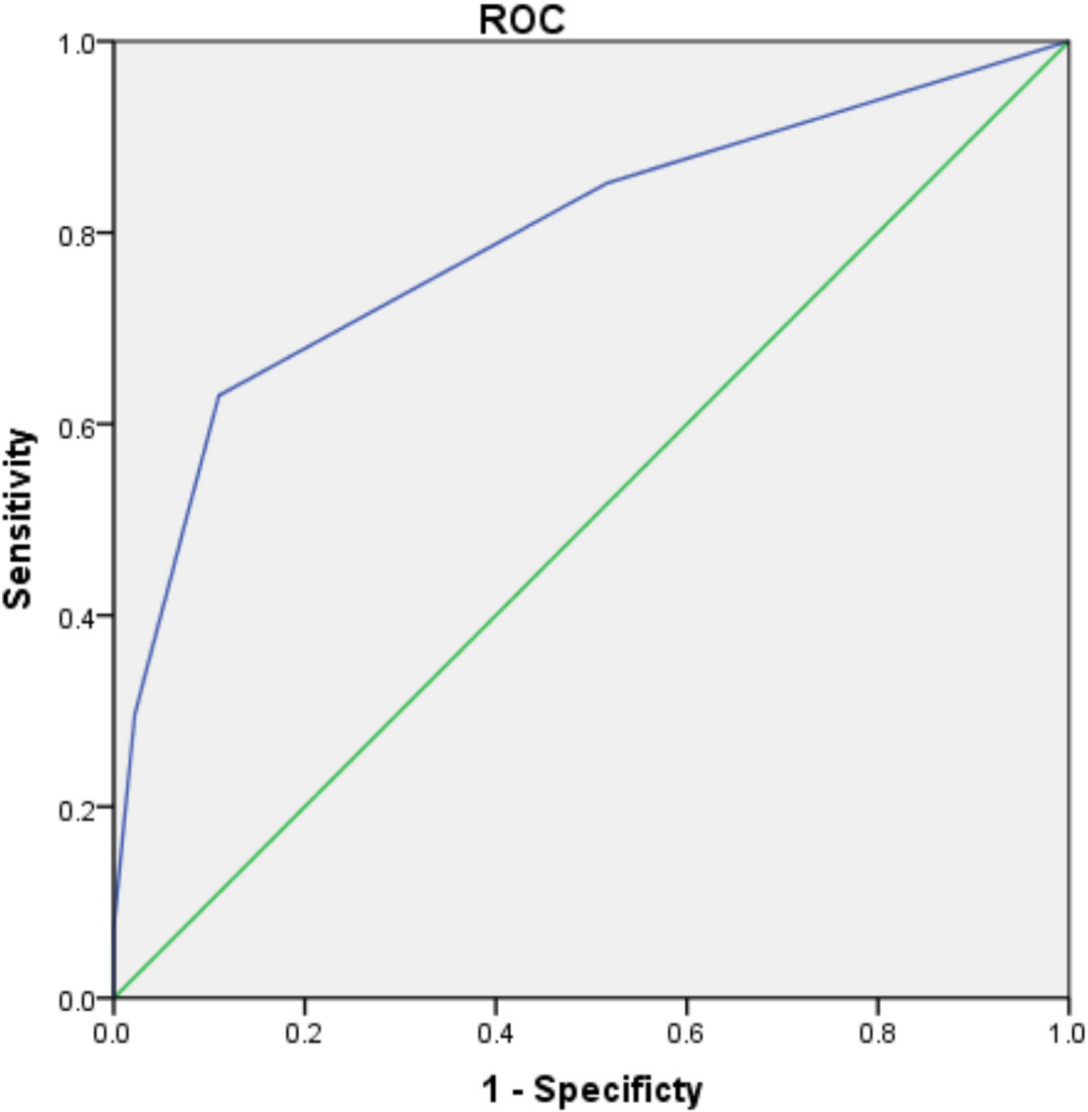
ROC curve of WCRDA in ruptured group

**Figure 2:**
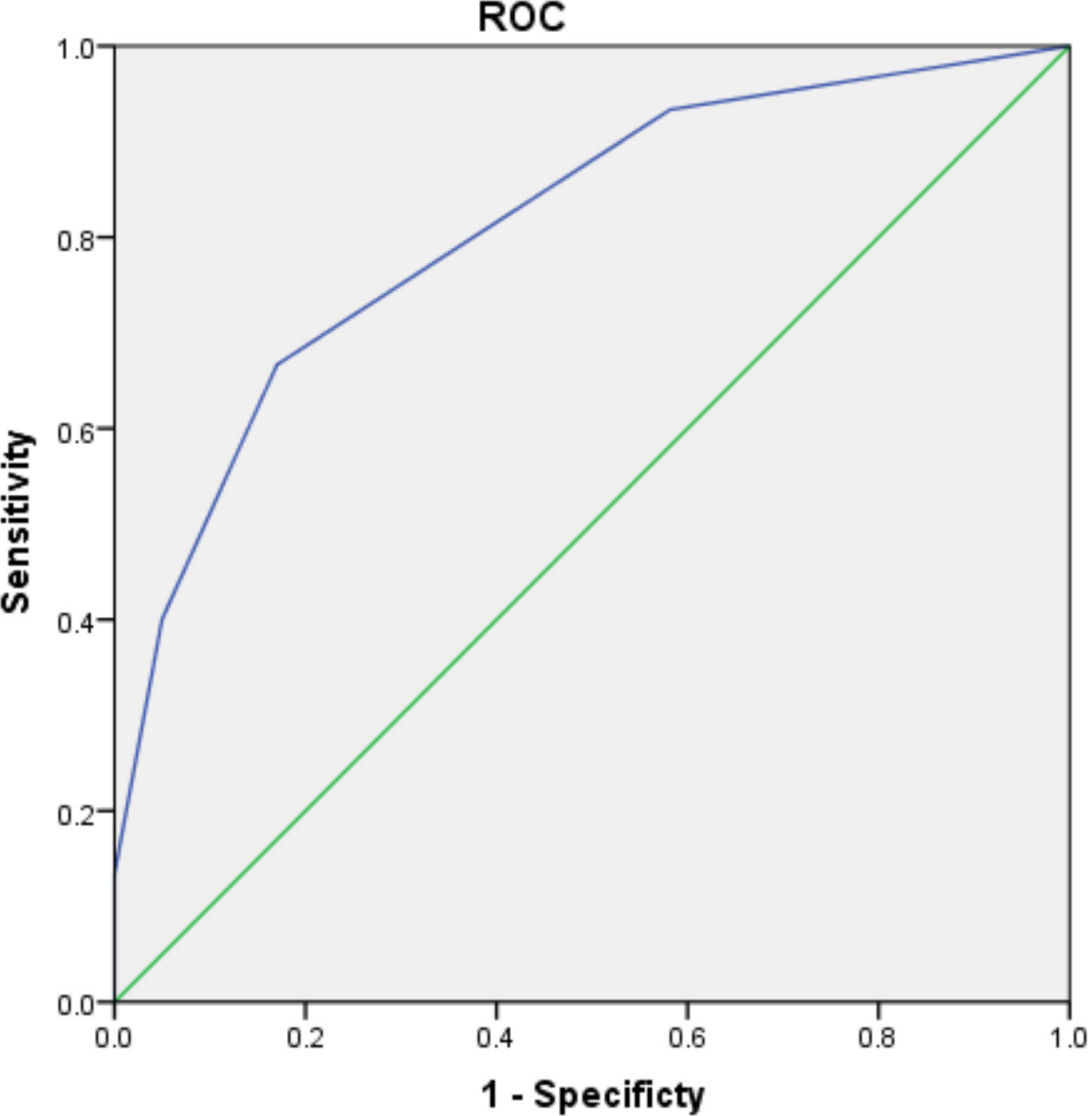
ROC curve of WCRDA in unruptured group

The ROC curves of the scoring system for predicting postoperative renal dysfunction are shown in Figures 1 and 2. The AUC of the ruptured group is 0.794 (95% CI 0.686–0.902, *P* < 0.001), and that of the unruptured group is 0.811 (95% CI 0.691–0.932, *P* < 0.001). The Hosmer–Lemeshow goodness-of-fit test was not statistically significant (chi-square = 1.376, df = 3, *P* = 0.711 vs chi-square = 0.512, df = 3, *P* = 0.916), indicating that the model was acceptable.

## DISCUSSION

OSR or EVAR is the only way to save the life of patients with emergency or ruptured AAA. OSR has some limitations for elderly people, patients with a poor circulatory or respiratory system, and those who cannot tolerate general anesthesia or surgery for a long duration. The continuous development in recent years have allowed for great improvements in endovascular interventional technology, and the numbers and types of stent grafts have continued to increase [9]. In addition, endovascular treatment for rAAA has shown an increasing trend [10–12]. Despite these advances, the treatment of aneurysms in emergency departments is still a challenge, especially in developing countries, because of the lack of supplies or equipment [13]. Therefore, OSR provides a suitable alternative as it does not require intravascular stent and detailed measurements of aneurysm. It reduces the duration of hypotension, allowing surgeons to save time and increasing the survivability of patients [9, 14]. During OSR, retroperitoneal hematoma can also be fully cleaned, which is beneficial to the recovery of gastrointestinal function postoperatively and prevents abdominal compartment syndrome. OSR can effectively reduce the incidence of postoperative gastrointestinal complications and surgical mortality. At the same time, it can clear retroperitoneal hematoma to prevent the occurrence of abdominal compartment syndrome, which decreases the risk of renal dysfunction postoperatively. Thus, the scope of its application is relatively wide [15]; however, it still needs to be studied.

Since our study reviewed 13 years retrospectively, the influence of study period was analyzed. There was no statistically significant difference between the first half and the second half, so we believe that time did not interfere with the study substantially. It's always been known than the baseline, the morbidity of complications and the mortality of patients with ruptured or unruptured aneurysms are significantly different. Despite its difference, the comparison of ruptured and unruptured AAAs has an extensive applicability and practical usage for clinical work. Abdominal aortic aneurysms may rupture in a short time, especially in cases of symptomatic threatened rupture.

The mortality rates and risk factors of these two groups were different. Given the proportion of patients with acute hemodynamic instability and low Hb level in the ruptured group, the effect of blood loss on renal function is indubitable. Intraoperative blood loss are the independent risk factor of both groups. During blood loss, hypoperfusion makes the renal function of the patients suffer from ischemia, hypoxia and ischemia-reperfusion. This finding provides a good explanation for the cause of renal dysfunction in AAA OSR. The difference is that hemodynamic instability presented prior to surgery in the rupture group, i.e., massive blood loss may obscure the patient's basic body situation.

The other three independent risk factors are preoperative creatinine, smoking and antihypertensive drugs. Preoperative creatinine is a controversial risk factor. For patients underwent emergency operation, it is obviously that the preoperative creatinine is not able to reflect patients’ baseline level. However, elevated preoperative creatinine may indicate postoperative renal dysfunction in rAAA patients. This might be an early warning signal, which may alert surgeons that perioperative renal protection is important for these patients. Surgeons could try to relieve the possible kidney ischemia or renal artery compression during the operation. This will be an interesting and useful finding despite further studies are needed. Some studies showed that smoking is independently associated with renal dysfunction and that increased inflammation causes changes in glomerular function. Moreover, smoking may lead to poor pulmonary function before general anesthesia, which can aggravate the burden of the kidneys [16–18]. A noticeable increase in renal dysfunction was observed for patients taking antihypertensive drugs in the unruptured group. The use of antihypertensive drugs may be related to renal dysfunction after OSR. Hypertension possibly causes damage to the kidneys, and patients who use antihypertensive drugs may have symptoms of hypertension.

There are no significant statistically difference between juxta-/supra renal AAA and postoperative renal dysfunction. Our technique performed in juxta-/supra renal AAA was to block the upper part of the abdominal aorta above renal artery and try to reduce clamping time. In principle, clamping time should be controlled within 25 minutes for each time. We surmised that short time clamping might not cause post-operational renal dysfunction, despite false negatives caused by insufficient data may also be the reason.

The mortality rate, ICU time, and renal dysfunction and complication rates of patients in the unruptured group were lower than those of patients in the ruptured group. Some studies referred to the possible strategies for renal protection. Doctors are accustomed to give rehydration to the patients with blood loss. However, excessive fluid infusion in the emergency department often reduces blood-clotting ability, thereby decreasing the thrombus around the site of injury. With the increase in local blood pressure, the protective spasm of the blood vessels is relieved, thereby worsening hemorrhage. Infusing a large amount of fluids does not increase renal perfusion. Instead, it increases the burden on the kidneys, and limited fluid resuscitation technology may play a positive role in emergency patients [19]. Avoiding excess fluid administration or limited fluid resuscitation is important during the whole perioperative period. A previous study [20] reported that remote ischemic preconditioning (RIPC) might be useful to improve renal and cardiac indices following major cardiac or vascular surgery. However, another prospective study [21] suggested that RIPC is not useful for kidney protection. Nevertheless, some specific postoperative renal function protection measures are available. The renal artery perfusion pressure is affected by the autoregulation of renal blood flow. Therefore, the mean arterial pressure needs to be controlled to a certain extent after AAA surgery. For patients without high abdominal pressure, stabilizing the average blood pressure above 80 mmHg can achieve better renal perfusion. For patients with increased abdominal pressure, especially in abdominal compartment syndrome, measuring intra-abdominal pressure, such as intragastric or bladder pressure, is recommended. The target mean arterial pressure should be intra-abdominal pressure above 80 mmHg. However, if the target intra-abdominal pressure is too high, it should be reduced to at least 80 mmHg or 65 mmHg, as reported in another study [22]. Increased urine volume does not mean improved renal function. However, in cases with low blood volume, the body lacks an effective mechanism to change the blood flow in the kidneys. Small doses of dopamine and diuretics may have a beneficial effect on renal perfusion and ischemia, although low-dose dopamine does not improve or protect renal function in severe acute renal insufficiency [23].

In contrast to S.W. Grant's study, we found that treated hypertension is a strong risk factor following AAA OSR, and risk factors are less related to age, respiratory disease, and other basic conditions or comorbidities according to our findings [7]. Smoking history is a new risk factor comparing with his, despite many current nephrology studies have pointed out that renal dysfunction is associated with smoking. Intraoperative factors, such as blood loss, may influence some factors that did not show strong performance in the regression analysis. Comparing with their research, we broadened the application scoping of the scoring system. Previous studies have found differences in epidemiological characteristics such as morbidity and mortality among patients with AAA of different countries or races. Their data were also based on Western populations. Thus, the heterogeneous population of patients [6, 24] should be considered, which needs to be verified in the future. Analyzing the outcomes of the patients, we can conclude that irreversible renal failure did not frequently occur. Therefore, renal dysfunction caused by ischemia or surgical strike is mostly transient.

A limitation is that it could not represent the Chinese or Asian population currently, despite a nearly 300 AAA OSR patients admitted to our institution during the last 13 years. Some risk factors have low positive rates, which may lead to false negatives. In addition, factors such as “blood loss” are not objective enough, but the result of logistics regression cannot be ignored. The impact on the accuracy of the scoring system needs to be examined. Regrettably, data on prognosis and renal dysfunction over 30-day are not included.

The WCRDA scoring system may help surgeons to predict renal insufficiency; however, its validity still needs to be verified by prospective or other institutional studies. Patients who are prone to have renal dysfunction need intervention, and appropriate treatment should be given.

## MATERIALS AND METHODS

The patients or their close relatives signed the informed consent and agreed that the data would be used for clinical research.

**Figure 3:**
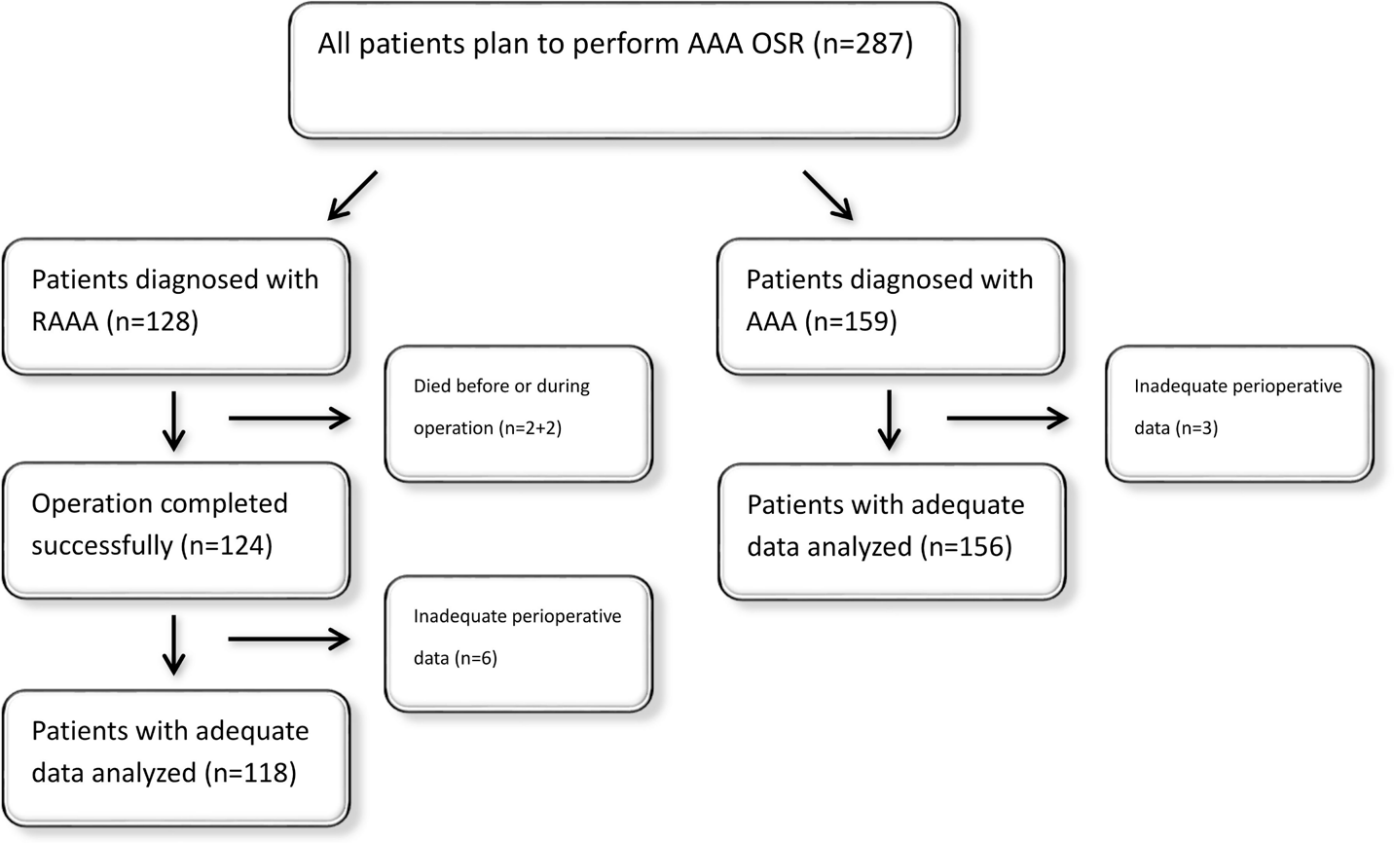
Study selection flow diagram

The patients in this study were recruited from the Vascular Department of West China Hospital, Sichuan University, between November 2003 and January 2017. As shown in Figure 3, in total, 287 patients were planned to perform AAA OSR. 274 patients who underwent OSR for AAA were included in this study finally. They were divided on the basis of ruptured (118) and unruptured (156) AAA. Cases of AAA were diagnosed and were measured via ultrasound, computed tomography, or magnetic resonance angiography and were proven after OSR. Patients with renal impairment (preoperative serum creatinine (SCr) >150 μmol/L) were not excluded because such condition could be considered an independent risk factor for postoperative renal dysfunction. Patients who died preoperatively or intraoperatively or who did not undergo OSR were all excluded.

As shown in Table 1, patient characteristics were as follows: Symptomatic AAA was defined as any symptoms may associated with abdominal aortic aneurysms ruptured. Hemodynamic instability was defined as a systolic blood pressure lower than 80 mmHg or as hemorrhagic shock diagnosed in the emergency department [25, 26]. Renal comorbidity was defined as preoperative SCr >150 μmol/L. Juxta-/supra renal AAA and AAA symptoms were confirmed by auxiliary examination and participating surgeons. Study period consist of first half and second half. Patients were equally divided into two groups according to the time of admission. When patients arrived at the hospital, their vital signs and blood test results were recorded. During surgery, the condition of aneurysms and operation information were recorded by the surgeon. The amount of blood loss is estimated by the surgeon according to the intraoperative condition and the amount of the storage of the aspirator. After the operation, creatinine levels at days 1, 3, and 5 and 48 h urine output were recorded. In this study, the SCr taken before intervention was defined as preoperative and baseline SCr.

In our study, we chose above or equal to stage 1 of Kidney Disease: Improving Global Outcomes criteria as the definition of renal dysfunction. Stage 1 encompasses an increase in SCr level of ≥0.3 mg/dL within 48 h or ≥1.5 times the baseline within 7 days, or a reduction in urine output (<0.5 mL/kg/h for 6 h) [27, 28]. Patients were labeled as renal replacement therapy needing one-time dialysis. Categorical variables were coded as 1 if they were positive recorded, and were coded as 0 if they were negative or not mentioned in the data base. For continuous variables the median was substituted for missing values. The variables missing for more than 15 percent of subjects were all excluded.

The WCRDA scoring system was created to describe the probability of postoperative renal dysfunction, and the receiver operating characteristic (ROC) curve was used to evaluate and examine its efficiency. The system is a simple scoring chart designed following logistics regression.

### Statistical analysis

Logistic regression analysis was used to identify risk factors for postoperative renal dysfunction. Candidate variables with *P* < 0.100 on univariate analysis were entered into the model. “Backward: Conditional” of logistic regression analysis was used to identify risk factors with statistically significant differences. The Hosmer–Lemeshow goodness-of-fit statistic was used to test the goodness of the scoring system. The ROC curve was used to evaluate the scoring systems for predicting renal dysfunction. An area under the curve (AUC) approaching 1 indicated perfect discrimination.

In all analyses, statistical significance was considered at *P* < 0.050. Statistical analysis was performed using IBM SPSS Statistics Version 22.0.

## Abbreviations

(AAA: Abdominal aortic aneurysm
(OSR: Open surgical repair
(rAAA: Ruptured AAA
(ROC: Receiver operating characteristic
(Hb: Hemoglobin
(WCRDA: West China renal dysfunction after AAA or rAAA OSR
(AUC: Area under the curve
(CI: Confidence interval
(OR: Odds ratio
(SCr: Serum creatinine
(EVAR: Endovascular aneurysm repair
(RIPC: Remote ischemic preconditioning
(ICU: Intensive care unit
